# Asymptomatic Miliary Tuberculosis in a Patient With Polymyalgia Rheumatica

**DOI:** 10.7759/cureus.90979

**Published:** 2025-08-25

**Authors:** Hirokazu Taguchi, Shuji Sumitomo, Hideki Oka, Shigeo Hara, Koichiro Ohmura

**Affiliations:** 1 Rheumatology, Kobe City Medical Center General Hospital, Kobe, JPN; 2 Pathology, Kobe City Medical Center General Hospital, Kobe, JPN

**Keywords:** asymptomatic, diagnosis, miliary tuberculosis, polymyalgia rheumatica, tuberculous meningitis

## Abstract

Miliary tuberculosis (TB), a severe form of TB caused by lymphohematogenous dissemination from a *Mycobacterium tuberculosis* focus, usually presents with systemic symptoms including fever and malaise. Here, we report the case of an 87-year-old woman treated with low-dose prednisolone and methotrexate for polymyalgia rheumatica, incidentally diagnosed with small miliary nodules in her lungs on chest computed tomography without any symptoms. Moreover, the patient reported being full of energy. The acid-fast bacterial culture and polymerase chain reaction test from various sites, including the transbronchial lung biopsy (TBLB) and bronchoalveolar lavage fluid, were negative; however, the histology of the TBLB specimen revealed epithelioid granuloma without acid-fast bacteria. One and a half months later, she was admitted to our hospital with fever and somnolence. The cerebrospinal fluid culture test was positive for *Mycobacterium tuberculosis,* and the patient was diagnosed with miliary TB complicated by tuberculous meningitis. This report suggests that although systemic symptoms usually accompany miliary TB, patients can be asymptomatic, and careful follow-up is important when suspected.

## Introduction

Globally, tuberculosis (TB) remains a major public health problem, causing millions of new cases and deaths each year. In this context, Japan is a low-endemic country for TB, with the number of new-onset TB cases declining and an incidence rate of 9.2 per 100,000 people in 2021 [1]. However, miliary TB, which results from the lymphohematogenous dissemination of *Mycobacterium tuberculosis*, accounted for 1.2% of all TB cases in Japan in 1988 and increased to 5.1% in 2022 [2]. This trend is likely associated with Japan's rapidly aging population, as individuals aged 60 and over constituted 92.8% (489/527) of miliary TB cases in 2022 [2]. In addition to advanced age, immunocompromised hosts are also important risk factors for miliary TB. These include autoimmune diseases, human immunodeficiency virus (HIV)/acquired immunodeficiency syndrome, cancer, and those receiving immunosuppressive agents [3]. Among the various immunosuppressive agents, glucocorticoid is an important risk factor. In a case-control study in the United Kingdom, the use of glucocorticoids, especially prednisolone (PSL) equivalents of 15 mg or more for one month, is a risk factor for developing TB [4].

Miliary TB symptoms are generally nonspecific and include fever, anorexia, and weight loss. Daily morning temperature spikes are reported to be a characteristic miliary TB symptom; however, occasionally, fever may be absent, and such cases may present with progressive wasting [3]. The diagnosis of miliary TB can therefore be challenging due to its insidious onset and nonspecific symptoms, often mimicking other febrile illnesses or malignancies. Moreover, many atypical cases, including those with acute respiratory distress syndrome, air leak syndrome, acute renal failure, and hepatic/gastrointestinal complications, have been reported; however, such cases usually develop more severe symptoms [3]. This diagnostic challenge is critical, as even a brief delay in diagnosis or treatment initiation can be associated with poor prognosis in a disease that is uniformly fatal if left untreated [3,5].

Here, we present the case of a completely asymptomatic patient with miliary TB and a typical miliary shadow revealed by chest computed tomography (CT), who subsequently developed TB meningitis one and a half months later. The complete absence of any symptoms when the miliary shadow was discovered on CT is particularly rare, presenting a significant dilemma for diagnosis and management. This case highlights the importance of maintaining a high index of suspicion and careful monitoring for suspected cases, even in low-endemic countries.

## Case presentation

**Figure 1:**
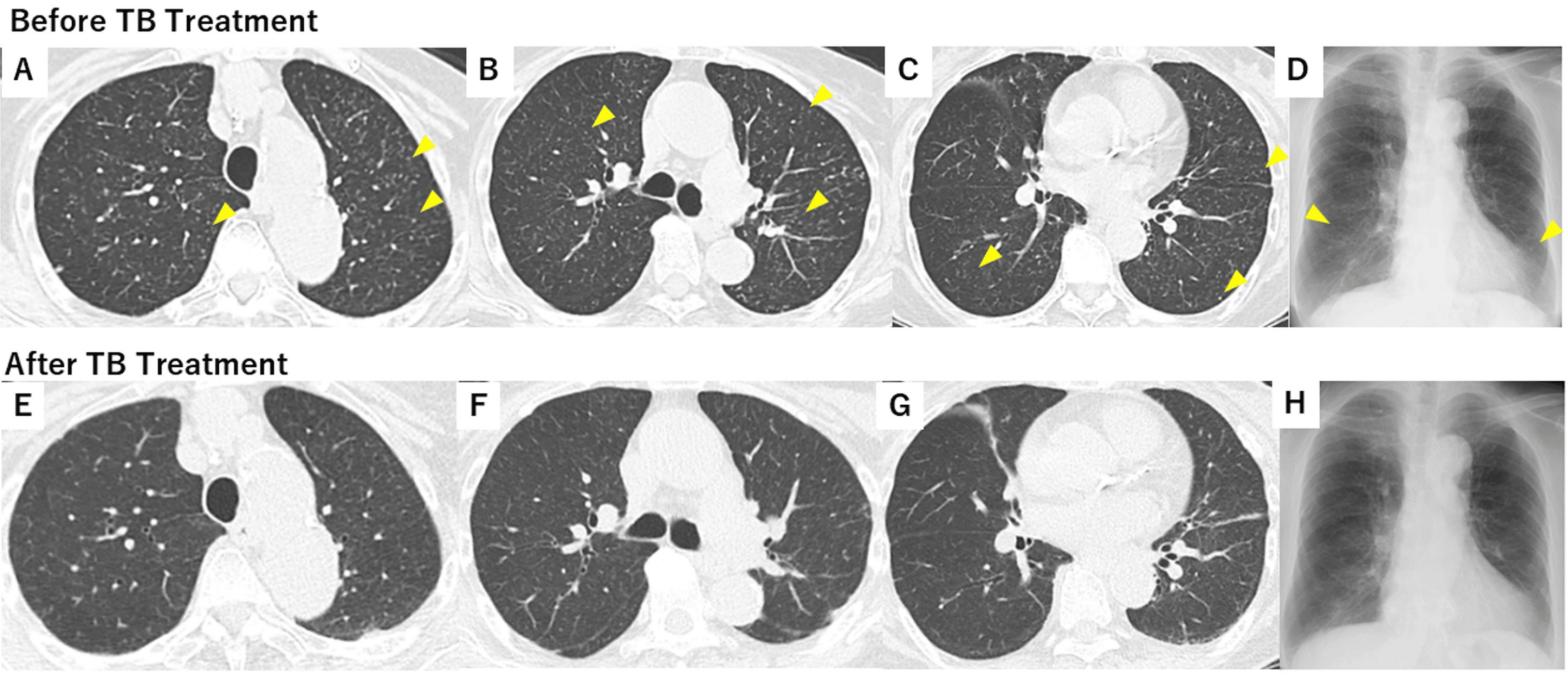
Chest CT and X-ray image before and after anti-TB treatment Chest CT and X-ray images were obtained one and a half months before admission (A-D) and five months after anti-TB treatment (E-H). CT slices were shown at the apex (A, E), pulmonary hilum (B, F), and lower lung field (C, G). Miliary nodules are distributed throughout the lungs (A-D), and only a portion of the numerous nodules are indicated by arrowheads. Those nodules have almost completely disappeared after anti-TB treatment (E-H). CT: computed tomography, TB: tuberculosis

An 87-year-old woman with a 14-day history of dyspnoea, tremor, and anorexia and a seven-day history of high-grade fever (≥38°C) was admitted to our hospital. She was diagnosed with polymyalgia rheumatica (PMR) two years prior to admission and started taking PSL and methotrexate (MTX), with maximum doses of 15 mg/day for PSL and 12 mg/week for MTX; her most recent doses were ≤5 mg/day of PSL and 6 mg/week of MTX. Prior to initiating glucocorticoid therapy for PMR, screening tests for TB, including serum interferon-γ release assay (IGRA) and chest X-ray, were negative. At an outpatient visit one and a half months prior, the patient had no symptoms and reported being full of energy despite a high C-reactive protein (CRP) level (4.41 mg/dL, reference: 0.00-0.50 mg/dL). On the same day, chest CT revealed miliary opacities in the lungs (Figures 1A-1C).

**Figure 2:**
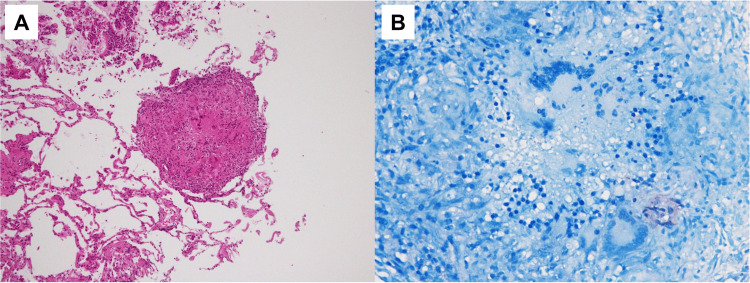
Histology of the lung biopsied one and a half months before admission Haematoxylin-eosin staining (A, original magnification ×100) and Ziehl-Neelsen staining (B, original magnification ×400).

Tests for IGRA and serum β-D-glucan were negative. Acid-fast bacterial culture tests of blood, sputum, urine, stool, and bronchoalveolar lavage fluid (BALF) could not detect acid-fast bacteria. Haematoxylin-eosin staining of the lung tissue obtained by transbronchial lung biopsy (TBLB) revealed an epithelioid granuloma with multinucleated giant cells without necrosis; however, no acid-fast bacilli were detected by Ziehl-Neelsen staining (Figure 2A-2B).

A polymerase chain reaction (PCR) test for *Mycobacterium tuberculosis* on lung biopsy tissue was not performed. The tentative diagnosis was drug-induced pneumonia due to the MTX treatment, which was discontinued. Three weeks before admission, she was afebrile, and her level of consciousness was normal at the outpatient visit. Physical examination at admission to our hospital revealed a blood pressure of 126/65 mmHg, a heart rate of 104 bpm (range 88-119 bpm, varying with body temperature), percutaneous oxygen saturation of 99% with ambient air, and a body temperature of 37.1℃. The Glasgow Coma Scale score was 15 points (full score), but her level of consciousness was unclear. Meningeal irritation and tremor were observed in both arms. Cranial nerve function tests, sensory and motor examinations, and reflexes were all normal. Cardiac murmur, lung crackles, lymphadenopathy, hepatosplenomegaly, arthritis, and dermatological abnormalities were not observed. Laboratory findings revealed a white blood cell count of 7,300/μL, a hemoglobin level of 12.5 g/dL, a platelet count of 23.5 × 104/μL, and a CRP level of 0.66 mg/dL (reference: 0.00-0.50 mg/dL) (Table 1).

**Table 1:** Laboratory findings on admission Alb: albumin, T-Bil: total bilirubin, AST: aspartate transferase, ALT: alanine transaminase, ALP: alkaline phosphatase, BUN: blood urea nitrogen, Cre: creatinine, CRP: C-reactive protein, KL-6: Krebs von den Lungen-6, ACE: angiotensin-converting enzyme

Complete blood count			Biochemistry					
Parameter	Result	Reference	Parameter	Result	Reference	Parameter	Result	Reference
WBC (x10^3^/μL)	7.3	3.9-9.8	Alb (g/dL)	2.8	3.9-4.9	K (mEq/L)	2.9	3.5-5.3
Neutrophil (%)	85.5	26.0-71.0	T-Bil (mg/dL)	0.6	0.2-1.2	Cl (mEq/L)	85	99-110
Lymphocyte (%)	9.5	19.0-61.0	AST (U/L)	30	8-40	Glucose (mg/dL)	225	70-110
Monocyte (%)	5.0	2.0-12.0	ALT (U/L)	16	8-40	CRP (mg/dL)	0.66	0.00-0.50
Haemoglobin (g/dL)	12.5	11.1-15.1	ALP (U/L)	179	100-340	KL-6 (U/mL)	454	0-499
Platelet Count (x10^4^ /μL)	23.5	13.0-37.0	BUN (mg/dL)	17.3	8-20	ACE (IU/L)	12.5	7.7-29.4
			Cre (mg/dL)	0.98	0.40-0.80	Lysozyme (μg/mL)	11.6	4.2-11.5
			Na (mEq/L)	121	136-148			

*Treponema pallidum* antibody and rapid plasma reagin tests were negative. The cerebrospinal fluid (CSF) test conducted on day 4 of hospitalization revealed an opening pressure of 7 mmH₂O. CSF analysis showed a white cell count of 393/μL with mainly polynuclear cells, a protein concentration of 216 mg/dL (reference: 15-45 mg/dL), a Cl level of 110 mEq/L (reference: 118-130 mg/dL), and a glucose level of 25 mg/dL (CSF to serum glucose ratio of 0.11) (Table 2).

**Table 2:** CSF findings on day 4 of hospitalization ADA: adenosine deaminase, Ag: antigen, RBC: red blood cell, N/A: not applicable, Cl: chloride, CSF: cerebrospinal fluid

Parameter	Result	Reference
Protein (mg/dL)	216	15-45
Glucose (mg/dL)	25	N/A
Cl (mEq/L)	110	118-130
Cell count (/μL)	393	0-5
Mononuclear (/μL)	127	N/A
Polynuclear (/μL)	266	N/A
RBC (/μL)	10	N/A
ADA (U/L)	16.5	N/A
β-D-glucan (pg/mL)	<6.0	<6.0
Cryptococcus-Ag	(-)	negative

**Figure 3:**
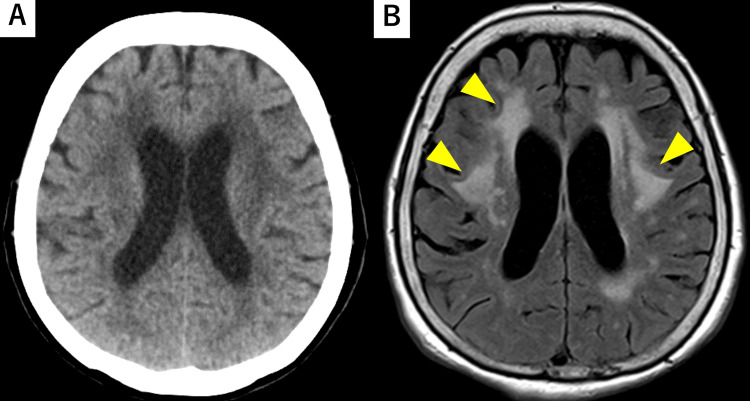
Head CT and MRI scan on admission Head CT (A) and FLAIR MRI (B) showing high signal intensity in the periventricular body of the lateral ventricle (yellow arrowheads). CT: computed tomography, FLAIR: fluid-attenuated inversion recovery, MRI: magnetic resonance imaging

CSF β-D-glucan and PCR for cytomegalovirus, herpes simplex, and herpes zoster were negative. Gram staining of CSF revealed no bacterial species. Head CT revealed no abnormalities (Figure 3A), but magnetic resonance imaging of the head revealed high signal intensity in the periventricular body of the lateral ventricle on fluid-attenuated inversion recovery (FLAIR) (Figure 3B).

**Figure 4:**
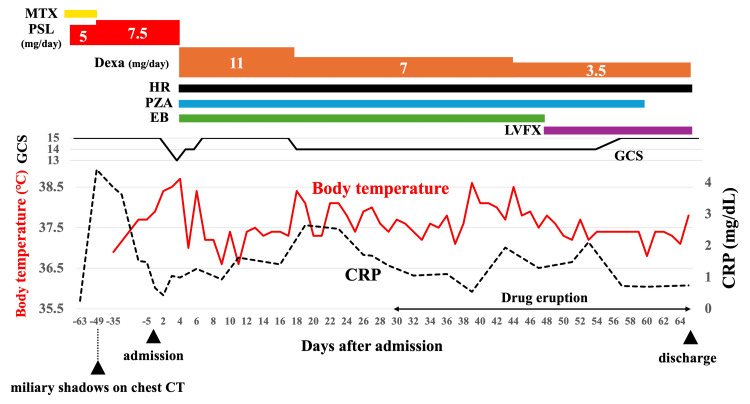
Clinical course of symptoms, CRP, and treatment MTX: methotrexate, PSL: prednisolone, Dexa: dexamethasone, HR: isoniazid and rifampicin, EB: ethambutol, PZA: pyrazinamide, LVFX: levofloxacin, GCS: Glasgow Coma Scale, CRP: C-reactive protein

The patient was diagnosed with subacute meningoencephalitis. She and her family did not have a medical history of TB, a recent travel history to countries with a high TB burden, or any other notable exposure history, including incarceration or living in crowded spaces like shelters. The ophthalmological examination revealed no significant findings. HIV testing was not performed, as the patient did not provide informed consent. Based on the chest CT findings, subacute disease course, and CSF findings, we decided to treat the patient for TB meningitis or *Listeria meningitis*. We initiated treatment with isoniazid, rifampicin, pyrazinamide, ethambutol, dexamethasone, and ampicillin. On day 9 of hospitalization, the CSF adenosine deaminase level was 16.5 U/L, and the bacterial CSF culture was negative. Therefore, ampicillin administration was discontinued. On day 11 of hospitalization, the CSF PCR for *Mycobacterium tuberculosis* was positive, and TB was later detected in the CSF culture, which was sensitive to all anti-TB drugs. A definitive diagnosis of TB meningitis was obtained, and miliary opacities of the lungs observed one and a half months ago on CT were suggestive of miliary TB. After TB treatment, the fever, consciousness, and tremors improved within about a week. Although the patient experienced a paradoxical reaction to TB treatment, including transient high-grade fever, elevation of CRP level, and deterioration of CSF parameters, and she also developed ethambutol-induced drug eruption, necessitating a switch to levofloxacin, her condition gradually improved thereafter. She was discharged after two months of TB treatment. Five months after discharge, her level of consciousness remained clear, and the miliary opacities of her lungs had improved (Figure 1E-1H). PMR remained inactive during TB treatment, with the PSL dose being 10 mg/day with concomitant rifampicin at the latest follow-up. The TB treatment regimens and the detailed clinical course, including the patient’s symptoms, CRP level, and treatments, are presented in Figure 4. TB treatment is planned to continue for 18 months.

## Discussion

Here, we present a case of miliary TB that was asymptomatic for at least one month, even after miliary shadows were detected on chest CT. A TB diagnosis was not obtained by culture or PCR of BALF and Ziehl-Neelsen staining of TBLB specimens from the lungs one and a half months before admission; however, the diagnosis was confirmed by culture and PCR of CSF specimens on admission. We performed a literature search using the PubMed database using the queries “miliary tuberculosis” OR “disseminated tuberculosis” AND “asymptomatic” from their inception date to August 30, 2024. The 61 retrieved papers were unrelated to asymptomatic miliary TB, suggesting that asymptomatic miliary TB has not been reported before. As miliary TB is a severe form of TB due to lymphohematogenous dissemination from a *Mycobacterium tuberculosis* focus [3], presentation with no symptoms for more than one month is unlikely.

This diagnosis of TB meningitis was confirmed in this patient; however, the direct link between the lung lesions and a disseminated TB infection remains debatable. Diagnostic criteria for miliary TB have been proposed previously to include [6]: (i) clinical presentation consistent with a TB diagnosis, including pyrexia with evening temperature increase, weight loss, anorexia, tachycardia, and night sweats for >6 weeks, responding to the anti-TB treatment; (ii) classical miliary pattern on chest radiograph; (iii) bilateral diffuse reticulonodular lung lesions on a background of miliary shadows on imaging; and (iv) microbiological or histopathological evidence of TB. In this case, only criterion (ii) was satisfied before the patient developed TB meningitis. Furthermore, the miliary lung lesions could be a sarcoid reaction to the drug or TB itself, as the epithelioid granuloma was not associated with necrosis. However, possible drug cessation, including MTX, did not ameliorate the lung shadow even after two months, and sarcoid reactions with such disseminated miliary shadows are rare. Nonetheless, the association between sarcoidosis and TB has long been discussed [7,8], and it is still a possible explanation for this case. The absence of PCR testing for the lung biopsy specimen may be a limitation of our case. Miliary TB is complicated by multiple organ involvement. Specifically, meningitis is reported in 10-30% of miliary TB cases, and one-third of TB meningitis cases have miliary TB [9]. In a retrospective study of 282 patients with miliary TB in China, 88% had central nervous system involvement [10]. Therefore, a miliary TB diagnosis for our patient is not surprising, even though culture or PCR tests were negative, considering the low positivity of culture tests in miliary TB (sputum, blood, gastric juice, spinal fluid, and BALF: 67.3% (69/101), 19.6% (19/97), 50% (5/10), 63% (29/46), and 71.1% (27/38), respectively) [11].

The typical symptoms of miliary TB include fever, chills, weight loss, anorexia, night sweats, fatigue, cough, and dyspnoea [3]. The reported frequencies of these symptoms are summarized in Table 3 [11-17]. However, miliary TB may not be diagnosed before death owing to the nonspecific symptoms and imaging findings, with diagnosis obtained at autopsy [18]. Thus, asymptomatic miliary TB is rare; however, two case reports have described present miliary TB cases with relatively mild symptoms [19,20]. One of the reasons for our patient being asymptomatic may be the hospital visits every two to four weeks for PMR management involving blood tests, including CRP, contributing to early detection of miliary TB. Thus, many patients with miliary TB may be asymptomatic if diagnosed at an early stage.

**Table 3:** Summarized frequencies of typical symptoms of miliary TB in the literature N/A: not applicable, TB: tuberculosis

References	n	Country	Age (mean)	Fever	Chills	Weight loss	Anorexia	Night sweats	Fatigue	Cough	Dyspnea
Kim et al. 1990 [12]	38	USA	60	89%	38%	66%	78%	76%	53%	55%	50%
Mert et al. 2001 [13]	38	Turkey	41	100%	N/A	100%	100%	100%	100%	68.40%	N/A
Wang et al. 2007 [14]	164	Taiwan	36.4	47.60%	N/A	7.32%	5.49%	3%	10.40%	22.60%	11%
Kerkhoff et al. 2017 [15]	410	South Africa	N/A	14.70%	N/A	43.80%	N/A	40.20%	N/A	47.10%	N/A
Mert et al. 2017 [11]	263	Turkey	44	100%	N/A	66.20%	84.80%	65%	90.50%	60.50%	30%
Wakamatsu et al. 2018 [16]	68	Japan	83	75%	N/A	N/A	64.70%	N/A	66.20%	27.90%	29.40%
Wei et al. 2024 [17]	288	China	42.6	77.10%	N/A	N/A	N/A	19.80%	N/A	54.50%	3.13%

## Conclusions

Asymptomatic miliary TB is rare but does exist. On coincidental detection of a miliary shadow on chest radiography or CT, miliary TB should not be ruled out. Furthermore, repetitive culture tests and careful monitoring are crucial for early detection and intervention. When miliary TB is strongly suspected, empirical anti-TB therapy should be initiated even in the absence of microbiological confirmation.
